# Development of Cyclodextrin-Functionalized Transethoniosomes of 6-Gingerol: Statistical Optimization, In Vitro Characterization and Assessment of Cytotoxic and Anti-Inflammatory Effects

**DOI:** 10.3390/pharmaceutics14061170

**Published:** 2022-05-30

**Authors:** Eman A. Mazyed, Farid A. Badria, Mai H. ElNaggar, Soha M. El-Masry, Sally A. Helmy

**Affiliations:** 1 Department of Pharmaceutical Technology, Faculty of Pharmacy, Kaferelsheikh University, Kaferelsheikh P.O. Box 33516, Egypt; 2 Department of Pharmacognosy, Faculty of Pharmacy, Mansoura University, Mansoura P.O. Box 35516, Egypt; badri002@mans.edu.eg; 3 Department of Pharmacognosy, Faculty of Pharmacy, Kafrelsheikh University, Kafrelsheikh P.O. Box 33516, Egypt; mai_elnaggar@pharm.kfs.edu.eg; 4 Department of Pharmaceutics, Faculty of Pharmacy, Damanhour University, Damanhour P.O. Box 22516, Egypt; Elmasry_soha@pharm.dmu.edu.eg (S.M.E.-M.); sallyhelmy@pharm.dmu.edu.eg (S.A.H.); 5 Department of Clinical and Hospital Pharmacy, Faculty of Pharmacy, Taibah University, Al Madinah Al Munawwarah P.O. Box 42313, Saudi Arabia

**Keywords:** gingerol, cyclodextrin, transethoniosomes, optimization

## Abstract

The poor solubility and stability of 6-gingerol (6-G) could hamper its clinical applications. The aim of the current study was to develop a novel ultra-deformable cyclodextrin-functionalized transethoniosomes (CD-TENs) as a promising delivery system for 6-G. Transethoniosomes (TENs) are flexible niosomes (NVs) due to their content of ethanol and edge activators (EAs). CD-functionalized nanoparticles could improve drug solubility and stability compared to the corresponding nanovesicles. 6-G-loaded ethoniosomes (ENs) were formulated by the ethanol injection technique in the presence and absence of EA and CD to explore the impact of the studied independent variables on entrapment efficiency (EE%) and % 6-G released after 24 h (Q_24h_). According to the desirability criteria, F8 (CD-functionalized transethoniosomal formula) was selected as the optimized formulation. F8 demonstrated higher EE%, permeation, deformability and stability than the corresponding TENs, ENs and NVs. Additionally, F8 showed higher cytotoxic and anti-inflammatory activity than pure 6-G. The synergism between complexation with CD and novel ultra-deformable nanovesicles (TENs) in the form of CD-TENs can be a promising drug delivery carrier for 6-G.

## 1. Introduction

**Figure 1:**
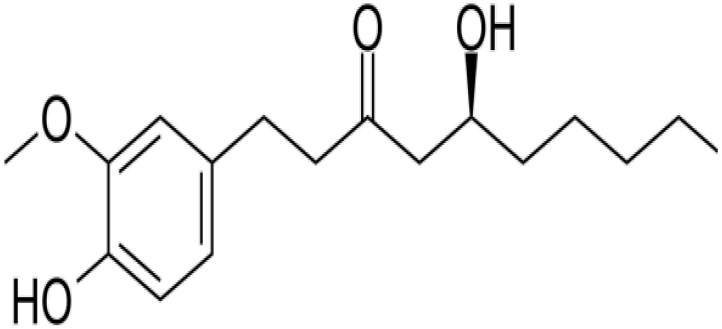
The chemical structure of 6-G.

Ginger (*Zingiber officinale Roscoe*) is a plant that has long been used as a food spice and medicinal herb, mostly to alleviate headaches, colds, nausea and vomiting [1]. Moreover, ginger has a number of beneficial medical properties, including antioxidant [2] and anti-inflammatory [3] and anticancer effects [4]. These biological activities have been associated with the main secondary metabolites of ginger (gingerols). Among the gingerols, 6-gingerol (6-G) is the most prevalent secondary metabolite in ginger oleoresin (Figure 1). 6-G could be suggested as a potential candidate for the treatment of cancer [5]. 6-G has been evaluated for its cytotoxic activity in various cancer cells, including colon cancer [6], cervical cancer [7], breast cancer [8] and prostate cancer [9]. Additionally, 6-G is an effective agent [10] that has been used for the management of chronic inflammatory diseases, such as osteoarthritis [11]. However, 6-G has poor aqueous solubility, low oral bioavailability and rapid metabolism [12]. These pitfalls hindered its medical applications. Cyclodextrin (CD)-based nanoparticulate system can effectively overcome these defects through the synergism between CDs and nanotechnology.

Nanocarriers could be used to provide significant enhancement in the solubility and stability of 6-G. In addition, nanoformulations can achieve targeted delivery and controlled release of drugs [13]. Conventional niosomes (NVs) are surfactant-based nanovesicular systems that could be effective carriers for both hydrophilic and hydrophobic drugs because of their amphiphilic nature. In addition, they are more chemically and physically stable than liposomes [14]. However, niosomal vesicles have low flexibility during permeation through the biological membranes.

Ethoniosomes (ENs) are a more flexible form of NVs due to their ethanol content, which can increase the fluidity of the vesicular membrane by intercalation into the lipid bilayer [15]. Additionally, the cholesterol content within ENs increases the stabilization of the bilayer membrane. Hence, ENs are more physically stable than spanlastics and can overcome various physical instability problems, such as vesicle aggregation and drug leakage [16].

The addition of edge activators (EAs) can improve the deformability and the capability of ethoniosomal vesicles to squeeze effectively through various biological membranes without loss of their intact integrity [17,18]. In this manuscript, the term transethoniosomes (TENs) was used to explore a novel ultra-deformable nanocarrier on the concept of using both ethanol and edge activators.

Despite the potential of nanoparticles in drug delivery, they possess some drawbacks, such as poor drug loading, low entrapment efficiency and instability. These problems related to nanoparticles could be overcome by using CDs. The development of CD inclusion complexes results in enhancing the loading, stability and solubility of poorly water-soluble drugs [19].

CDs are a group of natural cyclic oligosaccharides, such as αCD, βCD and γCD composed of six, seven or eight α-1, 4-linked D-glucopyranose subunits, respectively. CDs have a special geometric arrangement with a hydrophobic core and hydrophilic surface that permits the inclusion of hydrophobic drugs. Some researchers, such as da Silva et al. [20], showed that complexation with βCD has improved the solubility and cytotoxicity of 6-G. In another study, Pais et al. [21] prepared an effective inclusion complex of gingerol-enriched extract with γCD and reported that CDs are suitable carriers for gingerols.

This manuscript discussed the development of CD-functionalized TENs (CD-TENs) to investigate the effect of synergism between complexation with CDs and novel ultra-deformable nanocarriers (TENs) as a promising drug delivery system for 6-G.

## 2. Results and Discussion

### 2.1. Preformulation Study

#### 2.1.1. Docking Study

**Figure 2:**
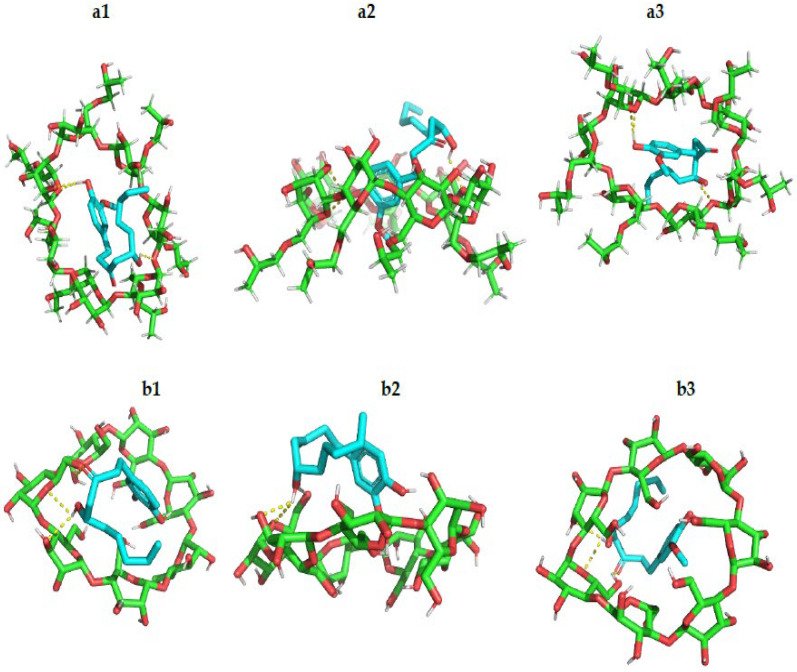
Three-dimensional molecular model stick representation of 6-G interaction within the cavities of (**a1**) HPβCD from top view, (**a2**) HPβCD from side view, (**a3**) HPβCD from basal view, (**b1**) βCD from top view, (**b2**) βCD from side view, (**b3**) βCD from basal view.

Molecular modeling methods are commonly applied to study the host–guest behavior of CD complexes [22]. A molecular docking study was conducted to predict the interaction of 6-G and its orientation within the cavities of β*CD* and HPβ*CD*. AutoDock Vina was used for calculating the binding energy of 6-G complexes with the two cyclic oligomers and comparing their stabilities. According to the docking results, 6-G showed better binding within the HPβ*CD* cavity with a binding energy of −4.6 kcal/mol, while the binding energy of 6-G complex with β*CD* was found to be −3.5 kcal/mol. The visualized docking poses (Figure 2) showed that 6-G can form three hydrogen bonds with the cavity atoms of both β*CD* and HPβ*CD*, while the structure of 6-G is more fitted into the cavity of the HPβ*CD*. These results indicated that the interaction of 6-G with HPβ*CD* is more favorable than its interaction with β*CD*. This could be explained by the more appropriate cavity size and the hydrophobic properties of HPβ*CD* pocket, which has additional hydroxypropyl residues. These residues help in stabilizing the 6-G guest molecule when it is sandwiched inside the cavity of HPβ*CD* [23].

#### 2.1.2. Phase Solubility Study

**Figure 3:**
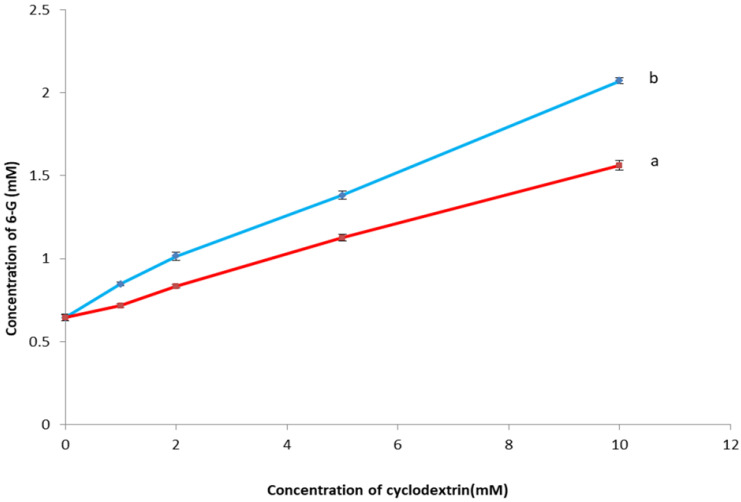
Phase solubility diagram of 6-G in the presence of increasing concentrations of (a) βCD and (b) HPβCD. **Abbreviation**: 6-G, 6-gingerol; βCD, beta cyclodextrin; HPβCD, hydroxy propyl beta cyclodextrin.

The phase solubility study can be used as a preliminary test for estimating the stoichiometry of the inclusion complex by determining the solubility of 6-G as a function of increasing concentrations of β*CD* and HPβ*CD*. Hence, it could assess the solubilizing efficiency of β*CD* and HPβ*CD* toward 6-G. Figure 3 demonstrated that the solubility of 6-G in water increased linearly, as the concentrations of both β*CD* and HPβ*CD* increased with slope values lower than unity. Such linear correlation is classified as an A_L_ type model, according to the pattern proposed by Higuchi and Connors [24]. The A_L_ type model suggested the possibility of formation of first-order complexes and that the stoichiometry of the 6-G inclusion complex is 1:1 in which a single guest molecule (6-G) is included into a single host molecule (β*CD* or HPβ*CD*) cavity.

6-G exhibited higher solubility in the case of HPβ*CD* than β*CD*. Additionally, the apparent stability constant (Ks) of β*CD* and HPβ*CD* complexes was 153.24 ± 2.16 and 248.05 ± 4.78 M^−1^, respectively. The complexation efficiency (CE) was 0.102 ± 0.024 and 0.161 ± 0.031, respectively. The sufficiently higher stability constant and complexation efficiency values demonstrated the development of a more stable inclusion complex between 6-G and HPβCD than β-CD.

According to the above results, HPβCD was chosen for the formation of the inclusion complex of 6-G in 1:1 molar ratio.

### 2.2. Evaluation of 6-G/HPβCD Inclusion Complexes

#### 2.2.1. Differential Scanning Calorimetry

**Figure 4:**
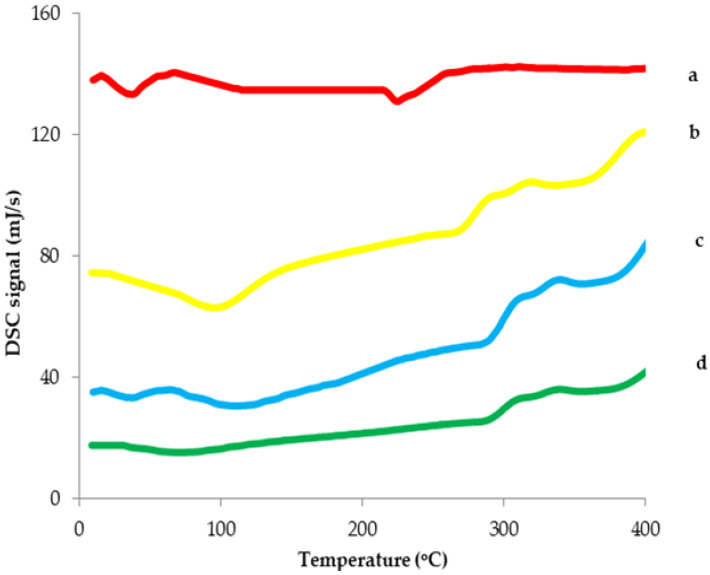
DSC thermograms of (a) 6-G, (b) HPβCD, (c) physical mixture, (d) 6-G/HPβCD complex. **Abbreviation**: DSC, differential scanning calorimetry; 6-G, 6-gingerol; βCD, beta cyclodextrin; HPβCD, hydroxy propyl beta cyclodextrin.

DSC study was performed in order to explore the interaction between 6-G and its host molecule (HPβCD) (Figure 4). When the guest molecule is trapped within CD cavity, its physicochemical parameters change, resulting in either disappearance or shift of its characteristic peaks to other temperatures. Therefore, the change of DSC thermogram could be used to investigate the formation of the inclusion complex. The DSC curve of 6-G exhibited two characteristic endothermic peaks at about 34.8 °C (∆H = 216.33 J/g) and 224.2 °C (∆H = 175.88 J/g) [20,21,25]. Other researchers, such as Pais et al. [21] and Singh et al. [25], studied the DSC profile of 6-G and found that it showed an endothermic peak at 29.5 °C and 31.23 °C, respectively, due to its melting point. Moreover, da Silva et al. [20] and Wei et al. [26] reported that 6-G exhibited an endothermic peak at 224.6 °C and 242.74 °C, respectively. In addition, HPβCD had a broad absorption peak at 91.9 °C (∆H = 290.51 J/g), which was correlated with the dehydration process in the HPβCD cavity and another peak at 262.2 (∆H = 155.25 J/g) that corresponded with the decomposition of HPβCD. The thermogram of HPBCD is in accordance with other researchers, such as Devine et al. [27], who stated that HPβCD demonstrated two endothermic peaks ranging from 25 to 103 °C, due to the dehydration, and 280 to 340 °C, due to the decomposition process. Cui et al. [28] reported that the DSC thermogram of HPβCD showed a broad endothermic peak at 89.9 °C, which is associated with loss of water. Meanwhile, the 6-G/HPβCD inclusion complex exhibited a new absorption peak at a higher temperature (285.3 °C, ∆H = 36.25 J/g) than that of HPβCD with the absence of the characteristic peaks of 6-G. The disappearance and the shift of endothermic peaks upon complexation with 6-G can be attributable to the change of the guest molecule after the formation of the inclusion complex and the replacement of water, present in the cavity of HPβCD, with 6-G. This is a clear confirmation of the development of the inclusion complex between HPβCD and 6-G.

The dehydration of HPβCD involves breaking different bonds between the HPβCD and water. It is then followed by the vaporization step of free water. The decrease in heat enthalpy of the inclusion complex is indicative of complexation of 6-G with HPβCD because the formation of inclusion complexes is associated with decrease in the heat enthalpy values due to the hydrophobic interactions between the HPβCD cavity and the guest molecule (6-G) [29].

Additionally, comparing the DSC thermogram of 6-G-loaded physical mixture with that of 6-G-loaded inclusion complex also confirmed the development of the inclusion complex between HPβCD and 6-G. In fact, the characteristic peaks detected for the individual components (6-G and HPβCD) were found in the physical mixture at 33.9 °C (∆H = 198.75 J/g), 103.75 °C (∆H = 288.39 J/g) and 270.5 (∆H = 160.46 J/g), while the absence and shift of these peaks in the thermal profile of the 6-G-loaded inclusion complex indicated proper molecular encapsulation of 6-G inside the HPβCD cavity [30]. The enthalpy change in the physical mixture was lower than that of the inclusion complex because the host–guest interactions were absent in the physical mixture [29].

These results are in agreement with da Silva et al. [20] who reported the disappearance of the characteristic peak of 6-G molecule after the formation of the inclusion complex with βCD. Moreover, Davaatseren et al. [31] found that after the development of the inclusion complex of cinnamaldehyde with βCD, the endothermic peak of cinnamaldehyde disappeared with the formation of a new peak with a different thermal transition. Pais et al. [21] reported that the complex of γ –CD with gingerols demonstrated a significant change in the DSC thermogram. The thermal peak associated with gingerol melting was not detected, and two new thermal events appeared.

#### 2.2.2. Thermogravimetric Analysis (TGA)

**Figure 5:**
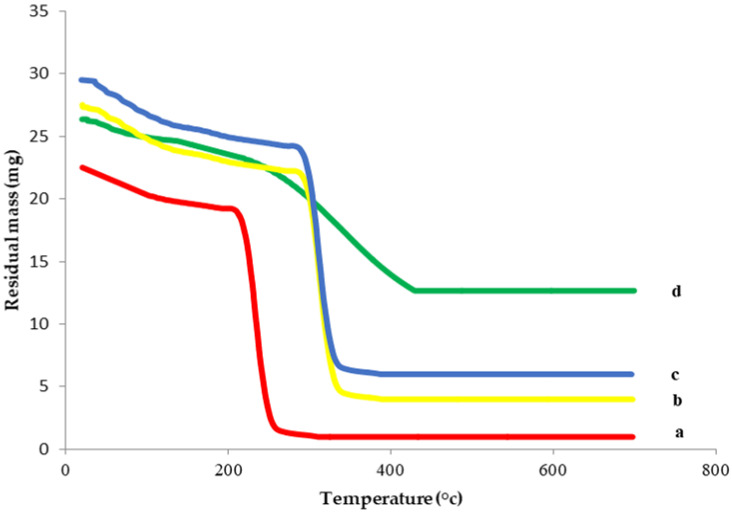
Thermogravimetric analysis of (a) 6-G, (b) HPβCD, (c) physical mixture, (d) 6-G/HPβCD complex. **Abbreviation**: TGA, thermogravimetric analysis; 6-G, 6-gingerol; βCD, beta cyclodextrin; HPβCD, hydroxy propyl beta cyclodextrin.

TGA is used for evaluating the thermal stability of different compounds that are related to dehydration, degradation and decomposition in response to temperature and time. The TGA spectra of 6-G, HPβCD and their inclusion complex were expressed in Figure 5. As shown, HPβCD had two stages of thermal weight loss. For the first stage, the weight loss (3.5%) was due to the vaporization of internal water [28,32]. The second stage was the apparent thermal weight loss caused by HP-β-CD decomposition, with 81.8% weight loss.

A comparison of the thermal weight loss of 6-G alone and within the physical mixture and the inclusion complex revealed that 6-G exhibited higher decomposition and a sharper weight loss (10.1% in the first stage and 88.7% in the second stage). Thermal analysis of the physical mixture exhibited a strong similarity to that of HPβCD, with 8.7% weight loss in the first stage and 57.6% in the second stage.

Meanwhile, the TGA spectrum of 6-G/HPβCD complex had a totally different thermal profile, which may be due to interaction with HPβCD within the inclusion complex. In addition, it showed higher thermal stability with a lower and more gradual thermal weight loss (1.8% in the first stage and 18.5% in the second stage). This might be explained on the basis of the thermal protection of 6-G following inclusion within HPβCD and the creation of chemical bonds between 6-G and HPβCD that were not destroyed easily with increasing temperature.

Both the DSC and TGA results established that the thermal characteristics of 6-G, as well as HPβCD, were changed after the development of the inclusion complex. In addition, the formation of the HPβCD inclusion complex could effectively improve the stability of 6-G.

### 2.3. Analysis of the 2^3^ Factorial Design

The optimization technique could determine the most appropriate values of various factors needed to generate high-quality formulations [25]. The effects of various independent variables on the properties of 6-G-loaded ENs are investigated in Table 1. The optimized 6-G-loaded ethoniosomal formula was chosen depending on maximizing both EE % (Y1) and Q_24h_ (Y2).

The output results of the factorial design of 6-G-loaded ENs are shown in Table 2. The signal-to-noise ratio was measured using an adequate precision value. For both responses, the values of adequate precision were greater than the desired value (4), indicating that the present model can effectively navigate the design space. The data from both responses (EE% and Q_24h_) fit the linear model well (R^2^ = 0.9545 and 0.9921, respectively). The developed equations are statistically valid and fit well with the available data, as evidenced by the high values of R^2^, pred. R^2^ and adj. R^2^ for both EE% and Q_24h_. The response value predictability was represented by the pred. R^2^. The difference between the pred. and adj. R^2^ is lower than 0.20. Hence, there is an acceptable harmony between them.

**Figure 6:**
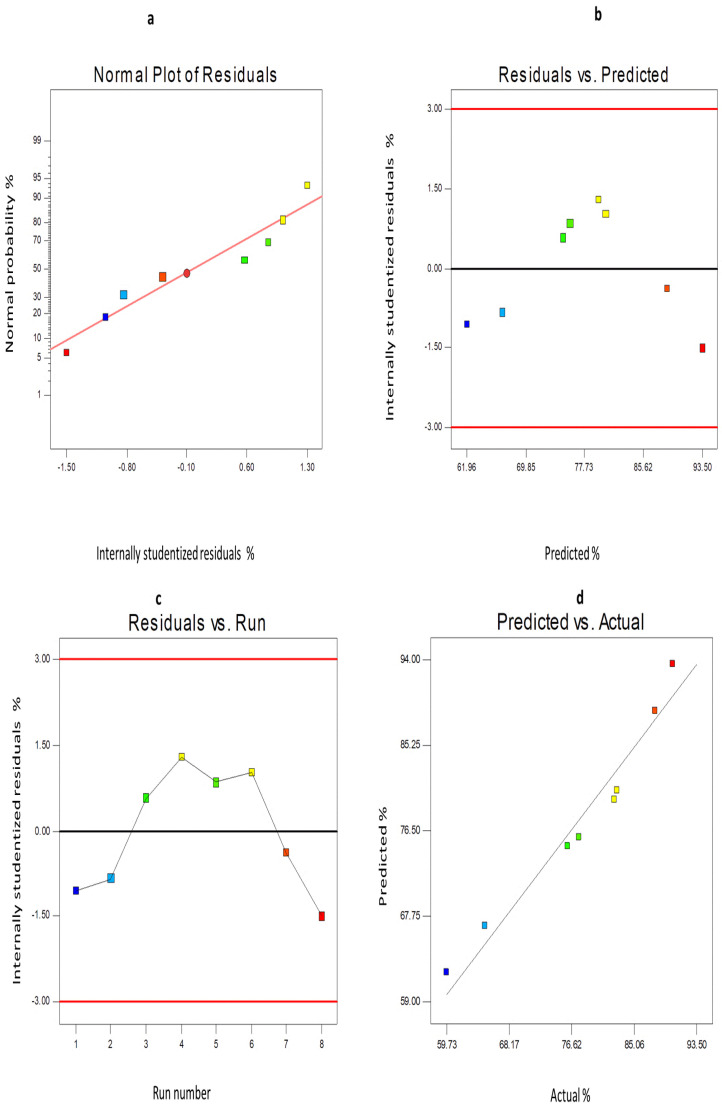
The diagnostic graphs for EE% of 6−G−loaded ENs (**a**) normal percentage probability plot of residuals, (**b**) internally studentized residuals versus predicted values graph (residuals versus predicted), (**c**) internally studentized residuals versus run number graph (residuals versus run), (**d**) predicted versus actual values graph (predicted versus actual). **Abbreviation**: 6−G, 6−gingerol; EE, entrapment efficiency of 6−G−loaded ENs; ENs, ethoniosomes.

**Figure 7:**
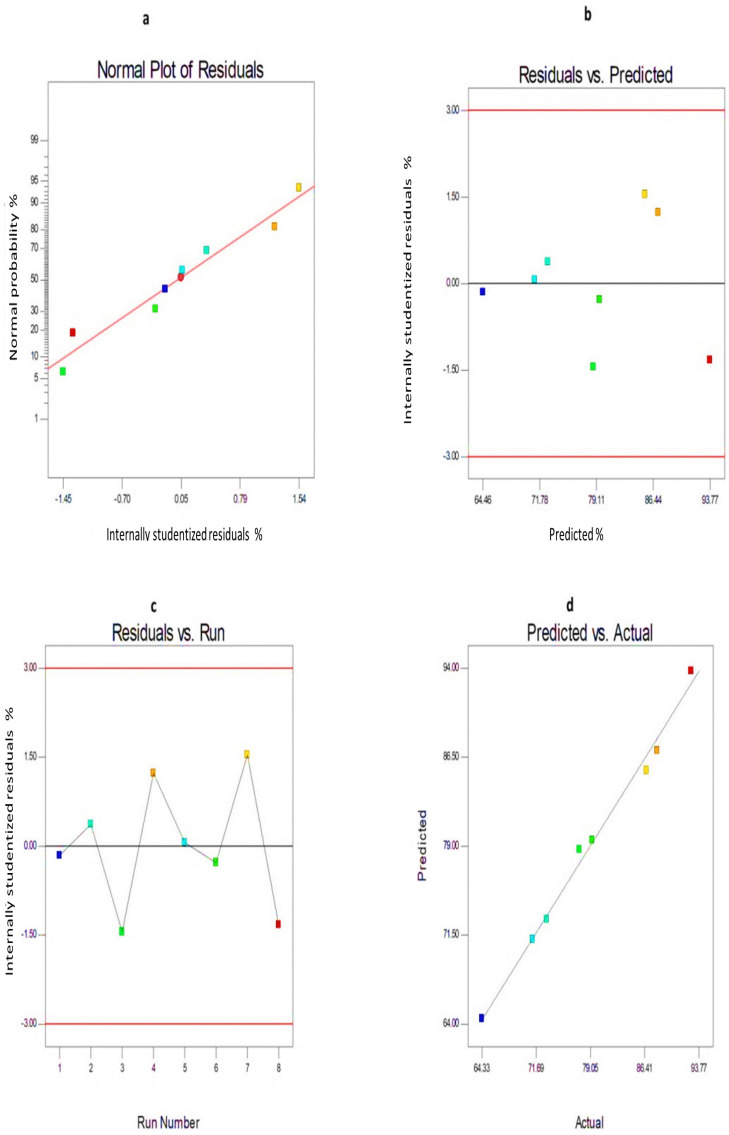
The diagnostic graphs for Q_24h_ of 6−G−loaded ENs (**a**) normal percentage probability plot of residuals, (**b**) internally studentized residuals versus predicted values graph (residuals versus predicted), (**c**) internally studentized residuals versus run number graph (residuals versus run), (**d**) predicted versus actual values graph (predicted versus actual). **Abbreviation:** 6−G, 6−gingerol; Q_24h_, % 6−G released after 24 h; ENs, ethoniosomes.

Furthermore, the diagnostic plots of both EE% and Q_24h_ were developed to assess the reliability and the reasonable fit of the present model (Figure 6 and Figure 7). The difference between the actual values of each response and their corresponding predicted values was used to determine the residuals (Y1 and Y2). Figure 6a and Figure 7a show the normal probability plots of the residuals as a linear pattern with a normal distribution of residuals, implying that the obtained data need no transformation. The colored points, demonstrating the values of both responses, were randomly distributed and presented around the zero axis (Figure 6b and Figure 7b), indicating the lack of constant error. Figure 6c and Figure 7c depict a uniform scattering of points, demonstrating the absence of lurking variables. The significance of the influence of the studied independent variables on Y1 and Y2 was demonstrated by the ANOVA analysis, as shown in Table 3.

#### 2.3.1. The Effect of Formulation Variables on EE% of 6-G-Loaded ENs

Table 1 demonstrates that the EE% of 6-G-loaded ENs was in the range of 59.73 ± 1.53 to 90.30 ± 1.47%. The drug content of 6-G-loaded ENs was in the range of 94.38–103.21%. The effect of the chosen independent variables on the EE% of 6-G-loaded ENs was depicted in Figure S1. Table 3 (ANOVA) demonstrates that the amount of Span 60 and the amount of CD had a significant effect on %EE of 6-G.

Regarding the amount of Span 60 (X1), it is clear that increasing the Span 60 amount had a considerable positive impact on the EE% of 6-G-loaded ENs (*p* < 0.01). This might be explained by improving the lipid bilayer’s stiffness and minimizing drug leakage [27].

With respect to the amount of HPβCD, it is worth noting that the formation of 6-G-loaded HPβCD inclusion complex had a significant (*p* < 0.01) positive impact on EE%. This may be explained on the basis of the capability of CDs to accommodate poorly water-soluble drugs within their cavity [19]. This is in agreement with Agüeros et al. [33] who reported that free paclitaxel has shown poor drug encapsulation within nanoparticles in comparison to its inclusion complex with HP-β-CD. Moreover, a study by Yuan et al. [34] demonstrated that the formation of ketoprofen β-CD inclusion complex enhanced the entrapment of ketoprofen within chitosan nanoparticles, especially in the case of substituted β-CD due to increasing the loading of hydrophobic drugs within the CD cavity.

#### 2.3.2. The Effect of Formulation Variables on Q_24h_ of 6-G-Loaded ENs

**Figure 8:**
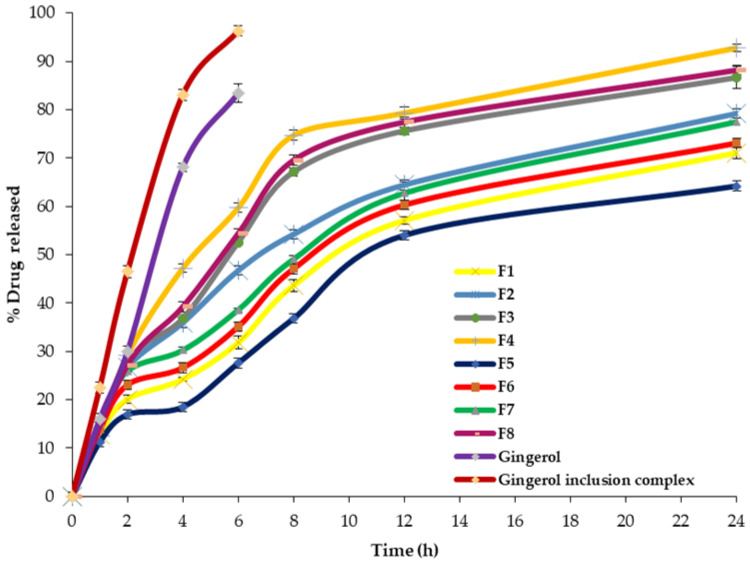
The in vitro release profile of 6-G-loaded ENs, 6-G/HPβCD complex and 6-G dispersion through cellulose membrane for 24 h.

The Q_24h_ of different 6-G-loaded ENs ranged from 64.33 ± 1.29 to 92.74 ± 1.54%, according to Figure 8. 6-G/HPβCD complex showed significantly higher drug release than 6-G. This could be explained on the basis of increasing the solubility of 6-G after inclusion in the HPβCD complex [20]. The release of 6-G from various ethoniosomal formulations was clearly more sustained than that of both 6-G and 6-G/HPβCD complex, which showed 60.03 ± 1.75% and 90.15 ± 1.42% drug released after 6 h, respectively. These findings suggested that the developed ethoniosomal formulations act as efficient reservoirs for 6-G, allowing it to be released in vitro for an extended period of time. Furthermore, the increased in vitro release of free 6-G and 6-G/HPβCD complex showed that the semipermeable cellulose membrane had no effect on the release of 6-G and that the sink conditions were successfully achieved [35]. These results agreed with Wei et al. [26] who reported that 6-G-loaded nanostructured lipid carriers demonstrated a more sustained release than free 6-G.

The impact of the selected independent factors on Q_24h_ is investigated in Figure S2. The amount of Span 60 (X1) had a significant negative effect on Q_24h_ of G-loaded ENs (*p* < 0.01). This could be due to increasing the stiffness of the vesicular bilayer, which reduces drug efflux from the ethoniosomal vesicles [14].

With respect to the amount of EA, it is clear that TENs have a significantly (*p* < 0.001) greater Q_24h_ than the corresponding ENs. This could be explained by the fact that larger levels of 6-G were released as a result of the combined effects of ethanol and EA [8,32], which resulted in more deformable vesicles [35]. The addition of EAs resulted in higher deformability of TENs that enabled them to squeeze and pass easily through narrow pores in the biological membranes [17,18].

Regarding the amount of HPβCD, it is obvious that it had a significant positive influence (*p* < 0.0001) on % drug released. The in vitro release of 6-G-loaded CD-TENs was higher than the corresponding TENs. This might be attributed to the loss of drug crystallinity, development of hydrogen bonds between the drug and CD and, accordingly, increasing the solubility of 6-G after inclusion within HPβCD [19,20]. These findings are comparable with those of Wang and Li et al. [36] who reported that the solubility and in vitro release of raloxifene increased after the formation of CD/Chitosan nanoparticles along with increasing drug bioavailability. Additionally, Dora et al. [37] revealed that there was an increase in the release and bioavailability of erlotinib when it was formulated in the form of erlotinib-CD/nanosponge complex.

The kinetic study (Table 4) demonstrated that the in vitro release of 6-G-loaded ENs followed the Baker–Lonsdale model and that of 6-G and 6-G/HPβCD complex fitted with the Hixson–Crowel model, as revealed by the correlation coefficient.

#### 2.3.3. The Optimization of 6-G-Loaded ENs

Using the Design-Expert software, the numerical analysis was performed to optimize 6-G-loaded ENs by maximizing both EE% and Q_24h_. The optimum ethoniosomal formula was chosen on the basis of the desirability criterion by optimizing many response factors at the same time. A desirability value is assigned to each response, and the total desirability value is calculated by taking the average of the individual desirability values. The overall desirability value ranges from 0 to 1, with 0 denoting a wholly undesirable response and 1 denoting an ideal response. Higher desirability values denote closeness to the target value [35,38].

The 6-G-loaded CD-TENs formula (F8) had the greatest values of desirability (0.925). Consequently, it was chosen as the optimized 6-G-loaded ethoniosomal formula. In addition, the predicted values of EE% and Q_24h_ were 90.10% and 88.63%, respectively, and a small % relative error (−0.22 and 0.65) was detected for EE% and Q_24h_, respectively. These findings backed up the ability of the present model to choose the optimal transethoniosomal formula (F8).

### 2.4. Characterization of the Optimized 6-G-Loaded TENs

#### 2.4.1. Morphological Characterization by SEM

**Figure 9:**
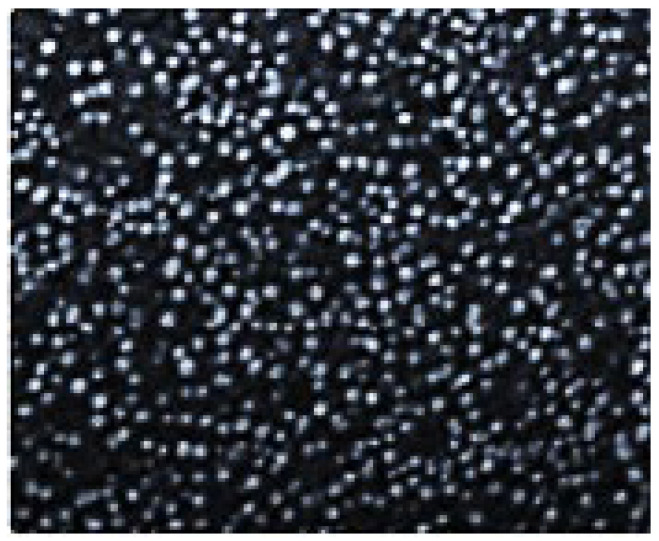
Scanning electron micrograph of the optimized 6-G-loaded CD-TENs. **Abbreviation:** 6-G, 6-gingerol; CD-TENs, cyclodextrin-functionalized transethoniosomes.

The SEM micrograph (Figure 9) describes the morphological characters of the 6-G-loaded CD-TENs (F8) as discrete and spherical nanovesicles. The spherical morphology of 6-G-loaded CD-TENs could be explained on the basis of the amphiphilic nature of Span 60 [39]. Accordingly, within the aqueous medium, a closed ethoniosomal bilayer would be formed that tends to minimize their surface free energy by the development of spherical transethoniosomal vesicles [14,40].

#### 2.4.2. Estimation of Vesicle Size and Zeta Potential

**Figure 10:**
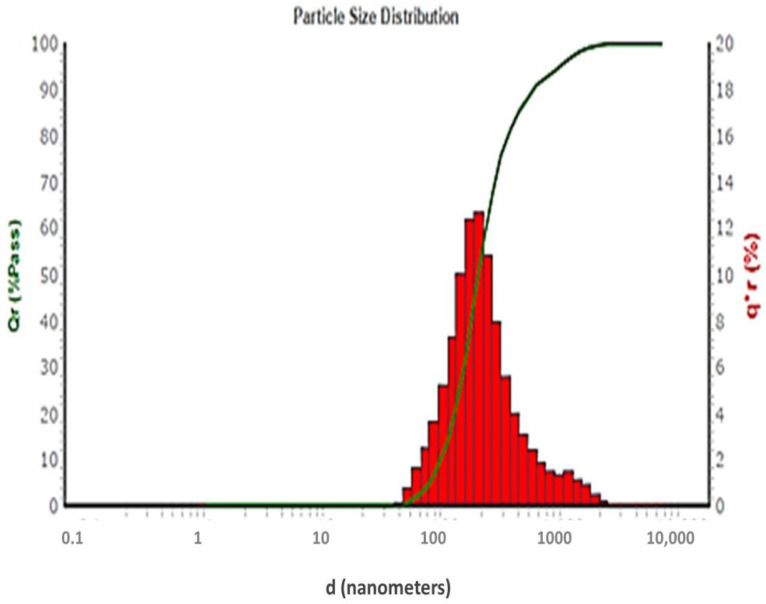
Particle size distribution curve of the optimized 6-G-loaded CD-TENs (F8).

Figure 10 exhibited the particle size distribution pattern of the optimized 6-G-loaded CD-TENs (F8). The vesicle size of the 6-G-loaded CD-TENs was 180.3 nm with a 0.382 polydispersity index (PDI) value that demonstrates the low particle size variation between different CD-functionalized transethoniosomal vesicles [41].

The zeta potential reflects the charge and the stability of the CD-TENs. A large value of zeta potential of F8 (+33.1 mv) indicates the stability of the nanodispersion of CD-TENs because of the repulsive force between the transethoniosomal nanovesicles and the presence of a high-energy barrier between them that inhibits their aggregation [42].

### 2.5. Comparative Study

#### 2.5.1. Measurement of Vesicle Elasticity

Traditional NVs are non-elastic nanovesicles that may rupture during permeation through the biological membranes [43]. The capability of CD-TENs to squeeze efficiently through the tiny holes of biological membranes without rupturing is described by their deformability. The calculated DI of the optimized 6-G-loaded CD-TENs (30.49 ± 0.81) was significantly higher than that of the comparable TENs (20.25 ± 0.78), ENs (14.33 ± 0.39) and NVs (1.76 ± 0.04). This could be attributed to the dual effect of EAs and ethanol. Ethanol could intercalate within the vesicular membrane of the CD-TENs and hence increase its fluidity [15]. EAs enhance the ability of CD-TENs to pass through the biological membranes without defeating vesicular integrity [17,18]. Accordingly, CD-TENs are considered to be ultra-deformable nanovesicles.

#### 2.5.2. Ex Vivo Intestinal Permeation Study

**Figure 11:**
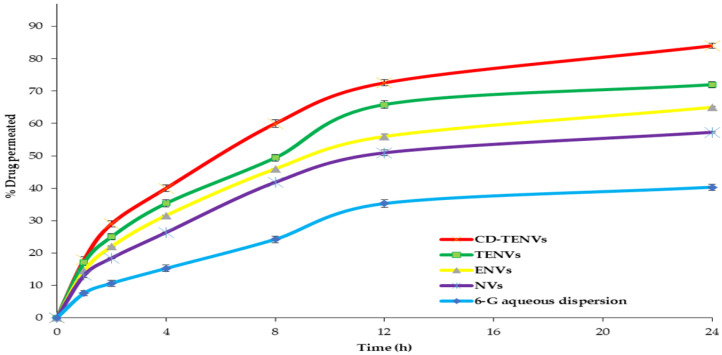
The ex vivo permeation profile of free 6-G, the optimized 6-G-loaded CD-TENs, TENs, ENs and the niosomal formulation for 24 h. **Abbreviation:** 6-G, 6-gingerol; CD-TENs, cyclodextrin-functionalized transethoniosomes; TENs, transethoniosomes; EN, ethoniosomes; NVs, niosomes.

The effect of encapsulating 6-G within CD-TENs on its permeability was examined by the ex vivo intestinal permeation study through the excised rat intestine (Figure 11). The optimized 6-G-loaded CD-TENs exhibited significantly higher permeation (84.15 ± 3.68%) in comparison to free 6-G dispersion (40.33 ± 1.89), the corresponding TENs (72.14 ± 2.66%), ENs (65.39 ± 1.21%) and NVs (57.26 ± 1.16%). Table 5 further reveals that the optimized 6-G-loaded CD-TENs, TENs, ENs and the niosomal formulation improved the flux of 6-G more than the 6-G aqueous dispersion, with enhancement ratios of 8.61, 7.20, 6.47 and 5.11, respectively.

The higher permeability of 6-G-loaded CD-TENs could be interpreted on the basis of the ultra-deformability of CD-TENs due to the presence of both ethanol and the EAs, which improved the deformability, and hence, permeability of the CD-TENs across various biological membranes by squeezing without rupture [16,44]. Additionally, improving the solubility of 6-G after inclusion within the HPβCD complex resulted in higher % 6-G permeated [32].

#### 2.5.3. The Stability Study

The stability of the nanoparticulate system is a vital matter of concern during shelf life. The stability test explored the effect of storage for 3 months at 4 °C on the drug content, EE% and Q_24h_ of different 6-G-loaded nanoformulations. Table 6 depicts the % change between the fresh and the stored formulations of 6-G-loaded CD-TENs, TENs, ENs and NVs. There was no significant change (*p* > 0.05) in the drug content, EE% and Q_24h_ of the HPβCD-functionalized TENs (F8). However, the corresponding NVs exhibited a significant decline in drug content (*p* < 0.01), EE% (*p* < 0.05) and Q_24h_ (*p* < 0.05) compared to the fresh formulation. Although the TENs and ENs exhibited no significant change (*p* > 0.05) in these properties, the % change in the drug content, EE% and Q_24h_ in the case of CD-TENs was significantly lower than that of the corresponding TENs and ENs.

A possible explanation for the improved stability of TENs using CDs might be the improved encapsulation of 6-G after the formation of the inclusion complex with HPβCD. In addition, Gadade and Pekamwar [19] reported that the balance developed between the hydrophilic and lipophilic functions and the steric interactions between alkyl chains in CD molecules could be a possible reason for increasing the stability of nanoparticles after using CDs. Chen et al. [45] demonstrated that CD-functionalized chitosan nanoparticles exhibited high stability at a temperature range of 10 to 70 °C. Baek and Cho [46] concluded that HP-β-CD is more efficient than hydroxy β-CD in enhancing the stability of paclitaxel-loaded solid lipid nanoparticles.

### 2.6. Biological Evaluation

#### 2.6.1. Cytotoxicity Assay

Cancer is a condition in which the body cells proliferate abnormally. According to the World Health Organization (WHO), 1 out of every 55 women in the world is diagnosed with breast cancer. This condition usually affects women over the age of 50. This disease has a significant mortality rate among women due to late detection and a lack of effective and safe treatments. In addition, chemotherapeutics have numerous undesirable effects due to their influence on the normal tissues [47]. Nanoparticles could be an effective technique for minimizing these negative effects.

**Figure 12:**
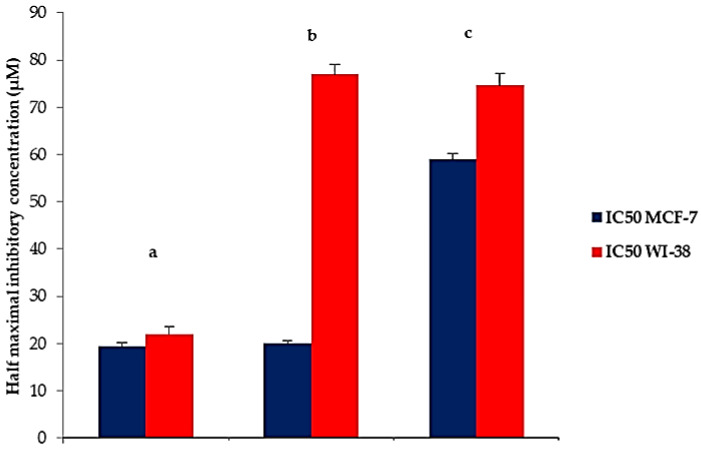
Cytotoxic activity, represented by IC_50_, against human breast cancer cell lines and normal lung fibroblast cells of (a) cisplatin, (b) F8 and (c) 6-G. **Abbreviations**: 6-G, 6-gingerol; IC_50_, the half maximal inhibitory concentration.

The optimized 6-G-loaded CD-TENs (F8) and 6-G were evaluated for their cytotoxic activity against human breast cancer cell lines (MCF-7cell lines) and the normal lung fibroblast cell lines (WI-38 cell lines) to calculate their IC_50_ and SI (Figure 12). The higher the magnitude of SI, the greater the selectivity of the cytotoxic agent [47].

Noticeably, the optimized 6-G-loaded CD-TENs (F8) showed significantly (*p* < 0.01) more potent cytotoxicity (IC_50_; 20.10 ± 0.51 µM) against MCF-7cell lines than pure 6-G (IC50;59.03 ± 1.12 µM).

There was no significant difference (*p* > 0.05) in the cytotoxic activity between F8 (IC50; 20.10 ± 0.51 mM and Cisplatin (IC_50_; 19.33 ± 0.85 µM). However, F8 had significantly (*p* < 0.05) higher selectivity (SI; 3.83 ± 0.11) in targeting breast cancer cells and less cytotoxicity (IC_50_; 77.12 ± 2.16 µM) on normal lung fibroblast cells compared to Cisplatin (SI; 1.13 ± 0.35, IC_50_; 22.04 ± 1.63 µM). The cell viability of a normal WI-38 cell line, after 48 h, increased significantly in the case of F8 (92.66 ± 0.94%) compared to both Cisplatin (5.02 ± 1.63%); *p* < 0.0001) and pure 6-G (77.03 ± 2.16%; *p* < 0.05).

The above results are in agreement with Zarei and Yaraghtala [48] who concluded that nanoliposomal gingerol had higher cytotoxicity than free gingerol, without any negative effects on other healthy body tissues. Additionally, Manatunga, et al. [49] found that hydroxyapatite-based nanoparticles containing 6-G produced more cytotoxicity on cancer cells with minimized effects on non-cancerous cells. In addition, Gadade et al. [19] demonstrated that CD-functionalized nanoparticles have the ability to decrease drug toxicity due to site-specific drug delivery. Chen et al. [50] reported that βCD gold nanoparticles of paclitaxel have a more effective targeted anticancer activity with lower toxicity on normal tissues.

#### 2.6.2. Cyclooxygenase Inhibition Activity

Cyclooxygenase-2 (COX-2) is the isoform of cyclooxygenase enzyme, which is responsible for the production of prostaglandins at the site of inflammation. The overexpression of COX-2 has been associated with chronic inflammatory diseases, such as rheumatoid arthritis [51]. The anti-inflammatory effect depends mainly on the inhibition of COX-2 enzyme, whereas the undesirable effects, such as gastrointestinal ulcers, are due to COX-1 inhibition because a number of physiological processes, such as protection of the stomach lining, are regulated mainly by COX-1-derived prostaglandins. In contrast, COX-2 is principally an inducible enzyme that is highly expressed in inflammatory conditions. Therefore, the selective COX-2 inhibitors that could effectively prevent the inflammation process without unwanted effect on physiological functions are more favorable.

**Figure 13:**
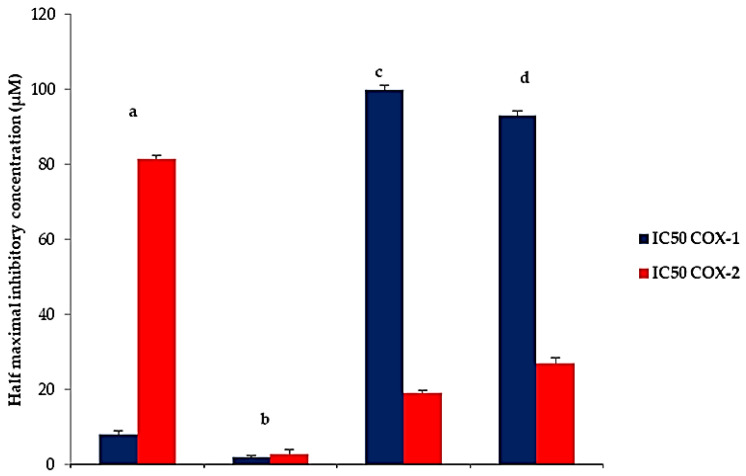
Cyclooxygenase inhibition activity, represented by IC_50_, against COX-1 and COX-2 enzymes of (a) AKBA, (b) indomethacin, (c) F8 and (d) 6-G. **Abbreviations**: 6-G, 6-gingerol; IC_50_, the half maximal inhibitory concentration; AKBA, 3-acetyl-11-keto-beta-boswellic acid.

The results of the COX inhibitory activity (IC_50_) (Figure 13) demonstrated that F8 showed significantly (*p* < 0.05) higher COX-2 inhibitory activity (IC_50_; 19.16 ± 0.62 µM) compared to 6-G (IC50; 27.04 ± 0.49 µM).

Additionally, F8 exhibited significantly (*p* < 0.001) higher COX-2 inhibitory effect and greater selectivity (IC_50_; 19.16 ± 0.62 µM, SI; 5.14 ± 0.08) than AKBA (IC_50_; 81.63 ± 1.47 µM, SI; 0.09 ± 0.003). With respect to Indomethacin, (IC_50_; 2.83 ± 0.96 µM, SI; 0.68 ± 0.04), F8 showed a lower COX-2 inhibitory effect but significantly (*p* < 0.01) higher COX-2 selectivity (SI; 5.14 ± 0.08). Consequently, F8 would be a safe anti-inflammatory agent that causes no side effects on the gastric mucosa and other physiological functions due to its high COX-2 selectivity.

The noticeable improvement in both the cytotoxic and anti-inflammatory activity could be attributable to the synergism between the HPβCD inclusion complex and TENs, which results in enhancing the solubility of 6-G and the elasticity of 6-G-loaded CD-TENs (da Silva et al., 2021). These results are comparable to Baskar et al. [52] who found that ultra-deformable nanovesicle of 6-G can enhance the biological permeability and release kinetics compared to pure drug. In addition, Sajeesh and Sharma [53] demonstrated that insulin/HP-β-CD-loaded nanoparticles had higher oral absorption and better drug delivery than the corresponding nanoparticles. Moreover, Gadade and Pekamwar [19] showed that CD-functionalized nanoparticles have a potential of increasing selectivity and reducing drug toxicity through targeted/site-specific drug delivery. Zhu et al. [54] reported that the development of indomethacin-CD nanoparticles resulted in site-specific delivery of indomethacin to intestinal tissues and, consequently, lower gastric irritation.

## 3. Materials and Methods

### 3.1. Materials

Silica gel (60–230 mesh) for column chromatography, silica gel 60 GF254 (20 × 20 cm, 0.2 mm thick) and aluminum sheets for thin-layer chromatography (TLC) were purchased from E. Merck (Darmstadt, Germany). Reversed-phase C18 silica gel (BAKERBOND^®^ octadecyl C18, 40 µm) was obtained from (J.T.Baker Inc., Philipsburg, PA, USA). Partisil KC18F Silica gel 60A with fluorescent indicator (5 × 20 cm, 200 µm layer thickness) and Vanillin/sulfuric acid spray reagent were purchased from Sigma Chemical Co. (St. Louis, MO, USA). The dry rhizomes of Zingiber officinale Roscoe were purchased from a local herbal market, Mansoura, Egypt, and authenticated in the Pharmacognosy Department, Faculty of Pharmacy, Mansoura University, by comparing them with the corresponding genuine samples. Sorbitan monostearate (Span 60), Polyoxyethylene (20) sorbitan monooleate (Tween 80), beta cyclodextrin (βCD, MW = 1134.98), 2-hydroxyl propyl beta cyclodextrin (HPβCD, MW = 1396, degree of molar substitution = 0.64), cholesterol (CHOL), Methanol (HPLC grade), N,N,N’,N’-tetramethyl-*p*-phenylenediamine (TMPD), 3-(4,5-dimethylthiazoyl)-2,5-diphenyl-tetrazolium bromide (MTT), 3-Acetyl-11-keto-beta-boswellic acid (AKBA) and Cisplatin were obtained from Sigma Chemical Co. (St. Louis, MO, USA). Sodium dodecyl sulphate (SDS), dipotassium monohydrogen phosphate, potassium dihydrogen orthophosphate, dimethyl sulfoxide (DMSO), petroleum ether, methylene chloride, ethyl acetate, *n*-butanol and absolute ethyl alcohol were obtained from El-Nasr Pharmaceutical Chemical Company (Cairo, Egypt). Celecoxib was donated by Pfizer pharmaceutical company (Egypt, under the authority of Pfizer, New York, NY, USA). Indomethacin was kindly provided by Medical Union Pharmaceutical Company (MUP Co., Abu Sultan, Ismailia, Egypt). Cellulose dialysis membrane (Spectra/Por^®^, 12,000 to 14,000 molecular weight cut-off) was obtained from Spectrum Laboratories Inc. (Rancho Dominguez, CA, USA). All other solvents and chemicals were of analytical grade and used as received.

### 3.2. Methods

#### 3.2.1. Isolation of 6-G

6-G was isolated from *Zingiber officinale* rhizomes. Briefly, *Zingiber officinale* rhizomes were powdered and extracted with methanol [55]. The crude methanolic extract was fractionated by a successive liquid–liquid partition with petroleum ether, methylene chloride, ethyl acetate and *n*-butanol. The methylene chloride fraction was subjected to column chromatography using normal-phase silica gel, and the sub-fractions containing gingerols were re-chromatographed over reversed-phase C_18_ silica gel to obtain the pure 6-G. The purity of 6-G was checked by TLC (Figure S3), and its structure was confirmed by co-chromatography with an authentic sample of 6-G previously isolated and characterized using ^1^H- and ^13^C-NMR spectroscopy in chloroform-d1 (CDCl_3_) (Figures S4 and S5).

#### 3.2.2. Preformulation Study

##### Docking Study

In silico docking study was conducted using Autodock vina (version 1.1.2) [56]. The structure data files for the host molecules (βCD/HPβCD) and for the 6-G were obtained from the PubChem database [57]. Autodock tools (version 1.5.6) were used for the preparation of different structures and converting them to the PDBQT formats. A grid box was established over the structure of the cyclic oligomer hosts with the dimensions of 40 × 40 × 40 and spacing of 0.375 Å, considering their entire structures as the active site. X, Y and Z coordinates of 25.138, –11.155, and 0.090 were used for βCD and 22.073, –10.831, and 2.853 for HPβCD. Other default parameters of Autodock vina were used. The docking scores obtained from AutoDock Vina were used as the binding free energy. Pymol [58] was used for the visualization of the docking poses with the highest docking score and the least root mean square deviation values (RMSD).

##### Phase Solubility Study

The phase solubility study was performed according to the method reported by Higuchi and Connors [24]. Excess amounts of 6-G were mixed in distilled water containing increasing concentrations (0–10 mM) of CD (βCD/HPβCD). The mixture was agitated for 72 h at 25 °C until equilibrium was attained using a magnetic stirrer (Jenway 1000, Jenway, UK). Afterward, the samples were filtered using a syringe filter with a nylon membrane (0.45 μm, Nylon Acrodisc, Gelman Sciences Inc., Ann Arbor, MI, USA), and the solubility of 6-G was determined using HPLC [20]. Triplicate measurements were performed, and the data were described as mean % 6-G released ± SD.

The HPLC study was carried out using methanol/water (80:20, *v/v*) as the mobile phase with a flow rate adjusted at 1.0 mL/min and 10 μL injection volume. The detection wavelength of 6-G was set at 278 nm. The concentrations of the 6-G standard calibration curve showed good linearity within the range from 20 to 100 μg/mL, with an R^2^ value of 0.9998. The HPLC chromatographic analysis was performed using the Thermo Scientific Dionex UltiMate HPLC system (Thermo ScientificTM, DionexTM, Sunnyvale, CA, USA) equipped with an autosampler (WPS-3000RS), a quaternary pump (LPG-3400RS), a column thermostat (TCC-3000RS) and a diode array detector (DAD-3000RS). The collection and processing of data were performed using Chromeleon 7 software. The chromatographic separation was performed using a reversed-phase C18 column (2.7 µm particle size, 150 mm × 4.6 mm i.d.).

The apparent stability constant (Ks) was calculated from the slope of the linear phase solubility graph of the 6-G/CD inclusion complex.

(1) K = slope/Intercept (1−slope)

The complexation efficiency (CE) could be calculated according to the following equation:

(2) CE= slope/(1−slope)

#### 3.2.3. Preparation of 6-G Inclusion Complexes

Inclusion complexes of the guest molecule (6-G) with the selected host CD molecule were prepared according to the solvent evaporation technique [59]. An accurate amount of 6-G was dissolved in 2 mL ethanol and added dropwise to the HPβCD aqueous solution (at the selected molar ratio). The mixture was magnetically stirred (Jenway 1000, Jenway, UK) for 2 h at 25 °C until a dried mass was developed. Then, the dried complex was pulverized, passed through sieve number 60 and stored in closed airtight containers.

#### 3.2.4. Evaluation of 6-G/HPβCD Inclusion Complexes

##### Differential Scanning Calorimetric (DSC) Study

The DSC study was performed to confirm the development of the inclusion complex between 6-G and the selected CD [20]. The thermal analysis of 6-G, the selected CD, the physical mixture and the developed inclusion complex was carried out using a DSC calorimeter (DSC-60, Shimadzu, Tokyo, Japan) within a nitrogen environment in a temperature range of 20 to 400 °C with a heating set of 10 °C/min in aluminum pans containing about 2 mg of the tested samples [21].

##### Thermogravimetric Analysis (TGA)

The thermal behavior of 6-G, the selected CD, the physical mixture and the developed inclusion complex was explored using a thermogravimetric analyzer (DTG-60, Shimadzu, Tokyo, Japan) by heating different samples in pierced aluminum-crimped pans over the temperature range of 20–700 °C with a heating rate of 10 °C/min and under nitrogen gas flow [60].

#### 3.2.5. Preparation of 6-G-Loaded ENs

6-G-loaded ENs were fabricated by the ethanol injection technique which is a repeatable and applicable method for the formation of small nanovesicles [16,35]. Eight 6-G-loaded ethoniosomal nanodispersions were fabricated in the presence and absence of EA (Tween 80) and CD to explore their influence on the EE% and Q_24h_ of 6-G within the ethoniosomal nanovesicles [61].

6-G (10 mg/mL), the non-ionic surfactant (Span 60) and CHOL (150 mg) were dissolved in ethanol (2 mL) until the development of a clear alcoholic solution. The alcoholic solution was further injected at a constant flow rate into a preheated (60 °C) aqueous phase in the presence or absence of EA (Tween 80). The beaker was properly covered to prevent the evaporation of ethanol. The dispersion was agitated continuously on a magnetic stirrer (Jenway 1000, Jenway, UK) until the formation of a milky dispersion (10 mL) of 6-G-loaded ENs. In order to obtain a reasonable vesicle size, 6-G-loaded nanovesicles were then sonicated for 10 min using a water-bath ultrasonicator (Elmasonic E 30 H, Elma, Singen, Germany). The 6-G loaded ENs were then kept at 4 °C overnight to attain complete maturation of the ethoniosomal nanovesicles. 6-G-loaded CD-ENs were formulated using the corresponding 6-G-loaded CD inclusion complex instead of free 6-G. The experimental runs and the components of different 6-G ethoniosomal formulations are investigated in Table 1.

The effect of the selected independent variables (the amount of Span 60, the amount of EA and the amount of CD) on different responses was explored by optimizing eight 6-G-loaded ENs using Design-Expert software (2^3^ factorial design) (Version 7.0.0, Stat-Ease, Inc., Minneapolis, MN, USA). Each variable was screened at two levels: the lower (−1) and the upper level (+1). The explored responses for 6-G-loaded ENs were the entrapment efficiency (EE %) and the percentage of 6-G released after 24 h (Q_24h_).

The diagnostic curves for the studied responses (EE% and Q_24h_) of 6-G-loaded ENs were plotted. The assessment of how well the current model described and predicted the experimental results was demonstrated by calculating the coefficient of determination (R^2^), predicted (pred R^2^) and adjusted R^2^ (adj R^2^). The significance level of the data of 6-G-loaded ENs was determined by the analysis of variance (ANOVA) according to the F-statistics (F-test) [62].

#### 3.2.6. In Vitro Characterization of 6-G-Loaded ENs

##### Determination of EE% of 6-G-Loaded ENs

The EE% of 6-G-loaded ENs was determined through separation of the free (un-entrapped) 6-G by the indirect technique using the ultracentrifugation method [16]. A 1 mL sample of the prepared 6-G ethoniosomal dispersions was centrifuged for 2 h at 15,000 rpm using a high-speed cooling centrifuge at 4 °C (Biofuge, primo Heraeus, Germany). The clear supernatant was then separated and filtered using a syringe filter with a 0.45 µm nylon membrane (Nylon Acrodisc, Gelman Sciences Inc., Ann Arbor, MI, USA). The amount of 6-G in the supernatant was analyzed using HPLC at 278 nm, and the EE% was calculated as follows:

(3) EE(%)=(Xt−Xs) × 100/Xt 

where Xt  =  total amount of 6-G, Xs  =  amount of 6-G in the supernatant

The drug content of 6-G (entrapped and unentrapped content) was estimated using HPLC at 278 nm via disrupting 1 mL of the 6-G-loaded ENs using 100 mL isopropanol [43,63].

##### In Vitro Release Study of 6-G-Loaded ENs

The in vitro release of 6-G from the fabricated ENs was tested using the membrane diffusion method [16] in which a glass cylinder was attached to the USP dissolution apparatus shaft (USP apparatus II, Erweka DT-720, Langen, Germany) [35]. A prehydrated semi-permeable cellulose membrane [64] was fixed carefully at the base of the glass cylinder between the donor and receptor compartments. The dissolution medium was 250 mL phosphate buffer (pH = 7.4) containing SDS (0.5% *w/v*) to ensure the attainment of sink conditions [65]. The receptor chamber was agitated constantly at 50 rpm and kept at 37 ± 0.5 °C to simulate the biological conditions. A 1 mL aliquot of the 6-G loaded ENs containing the entrapped drug was located in the donor compartment over the cellulose membrane. A 0.2 mL aliquot was withdrawn from the receptor compartment at predetermined time intervals for 24 h and replenished by an equal volume of the fresh phosphate-buffered solution to maintain a fixed volume of the dissolution medium [66]. The withdrawn aliquots were filtered using syringe filter with 0.45 µm nylon membrane (Nylon Acrodisc, Gelman Sciences Inc., Ann Arbor, MI, USA) and analyzed by HPLC at 278 nm for the content of 6-G released. Triplicate measurements were performed, and the data were described as mean % 6-G released ± SD.

#### 3.2.7. Statistical Optimization of 6-G-Loaded ENs

The optimized 6-G-loaded ethoniosomal dispersion was estimated using the Design-Expert software on the basis of estimating the overall desirability function, which represents the closeness of the studied responses (EE% and Q_24h_) to their optimal values [67]. The desirability criteria of the present model were based on maximizing both responses. The optimized ethoniosomal formula, which had the highest desirability value, was determined and validated by calculation of % relative error of both EE% and Q_24h_ as follows [68]:

(4) $$\%\;\mathrm{Relative}\;\mathrm{error}=\frac{\left(\mathrm{predicted}\;\mathrm{value}-\mathrm{observed}\;\mathrm{value}\right)*100\;}{\mathrm{predicted}\;\mathrm{value}}$$

Further characterization tests were then conducted on the selected 6-G-loaded ethoniosomal formulation.

#### 3.2.8. Characterization of the Optimized 6-G-Loaded CD-TENs

##### Scanning Electron Microscopy (SEM)

The morphological properties of the optimized 6-G-loaded ENs were described by SEM (Scanning electron microscope, JSM 6100 JEOL, Tokyo, Japan). An amount of 0.1 mL of 6-G-loaded ENs was suitably diluted by deionized water (10 mL). One drop of the diluted dispersion of 6-G-loaded CD-TENs was located carefully onto the SEM specimen stub using carbon double-sided tape. The ethoniosomal sample was then dried properly before being scanned with SEM [69].

##### Vesicle Size and Zeta Potential Estimation

The vesicle size and zeta potential of the optimized 6-G-loaded CD-TENs were estimated for describing the colloidal behavior of the ENs. A 1 mL aliquot of the optimized ENs was appropriately diluted with 200 mL of deionized water. The vesicle size and zeta potential of the optimized 6-G-loaded CD-TENs were measured in triplicate at 25 °C using NICOMP 380 ZLS zeta potential/particle sizer (PSS Nicomp, Santa Barbara, CA, USA) at 90° scattering angle [41].

#### 3.2.9. Comparative Study

##### Measurement of Vesicle Elasticity

The elasticity of the optimized 6-G-loaded CD-TENs was explored in terms of the deformability index (DI) using the following equation:

(5) $$\mathrm{DI}=J\;{\left(r_{v}/r_{p}\right)}^{2}$$

where J is the amount of the extruded 6-G-loaded CD-TENs, r_v_ is the vesicle size of the optimized 6-G-loaded CD-TENs (after extrusion), and r_p_ is the pore size of the nylon membrane filter.

The DI of optimized 6-G-loaded CD-TENs was determined by the extrusion technique through a 100 nm nylon membrane filter for 5 min [70]. The deformability of the optimized CD-TENs was compared with the corresponding transethoniosomal, ethoniosomal and niosomal formulations to explore the influence of CD, EA and ethanol on the elasticity of CD-TENs.

##### Ex Vivo Intestinal Permeation Test

The ex vivo permeation test was performed in order to explore the role of CD, EA and ethanol on the permeability of 6-G. The ex vivo intestinal permeation test was performed according to the ethical guidelines [71,72,73], and the test protocol was approved by the ethics committee (approval number KFS-2021/11) of the Faculty of Pharmacy, Kafrelsheikh University, Egypt. The rats (200–220 g male Wistar rats; *n* = 6) were sacrificed under anesthesia. The small intestines of rats were removed carefully and cleaned thoroughly using 0.9% saline solution to eliminate any unwanted mucosal content [74,75]. The excised small intestines were cut in the form of small sacs. The intestinal sacs were packed with the studied samples (1 mL), and the two edges of the sac were tied using a surgical thread. The tied intestinal sacs were fixed to the dissolution apparatus shafts [74,76]. The ex vivo intestinal permeation test of 6-G-loaded CD-TENs was carried out using 250 mL phosphate buffer (pH = 7.4) containing SDS (0.5% *w/v*) as the dissolution medium [12]. The receptor compartment was kept at 37 °C ± 0.5 °C and stirred at 50 rpm. A 0.2 mL sample was withdrawn at the predetermined time intervals (1, 2, 4, 8, 12 and 24 h) and replenished regularly by the same volume of fresh phosphate buffer. The withdrawn samples were filtered using a syringe filter with a nylon membrane (0.45 µm pore size, Nylon Acrodisc, Gelman Sciences Inc., Ann Arbor, MI, USA). The amount of 6-G permeated was determined using HPLC at 278 nm. The ex vivo permeation test was performed in triplicate, and the % 6-G permeated was estimated as the average ± SD. The ex vivo permeation of 6-G-loaded CD-TENs was compared with the free 6-G aqueous dispersion, the corresponding transethoniosomal, ethoniosomal and niosomal formulations. Additionally, the ex vivo permeation profile of the studied 6-G-loaded formulations was also compared by calculating the pharmacokinetic parameters [43], such as the steady-state flux (J_ss_), the permeability coefficient (K_P_) and the enhancement ratio (ER).

##### The Stability Test

The optimized 6-G-loaded CD-TENs and the corresponding TENs, ENs and NVs were kept for three months in firmly closed vials at 4 °C [77]. The studied formulations were compared with regard to their drug content, EE% and Q_24h_ to inspect the effect of the addition of CD, EA and ethanol on the stability of 6-G-loaded CD-TENs.

#### 3.2.10. Biological Evaluation

##### Cytotoxicity Assay

MCF-7 is a well-known human breast cancer cell line that has progesterone, estrogen and glucocorticoid receptors [78]. The MCF-7 cells were seeded at 5 × 10^4^ cells/mL in a 96-well plate (100 μL/well). After overnight incubation of the cells at 37 °C and 5% CO_2_, serial dilutions of the tested compounds or cisplatin (positive control) (50, 25, 12.5, 6.25, 3.125 or 1.56 M) were applied. As a negative control, 0.5% DMSO was utilized. The cells were incubated for 48 h. The cells were then incubated for another 4 h after the addition of 10 μL MTT and 5 mg/mL phosphate-buffered saline (PBS). Then, in order to solubilize formazan crystals, 100 L of acidified SDS solution was added. The 96-well plate was incubated at 37 °C with 5% CO_2_ for an additional 14 h. The absorbance was measured at 570 nm using a Biotech plate reader. The IC_50_ value was determined as the concentration that causes 50% inhibition of cell growth [55,79].

The selectivity of the tested active compounds toward breast cancer was evaluated by using normal lung fibroblast cells (WI-38) as a non-cancerous cell line. WI-38 cell lines were incubated with serial dilutions of the studied components. After 48 h of incubation, the viability of normal cells was checked as mentioned before. The selectivity index (SI) was calculated as follows [80]:

(6) $$\mathrm{SI}=\frac{\mathrm{IC}50\;\mathrm{in}\;\mathrm{normal}\;\mathrm{cell}\;\mathrm{line}}{\mathrm{IC}50\;\mathrm{in}\;\mathrm{cancer}\;\mathrm{cell}\;\mathrm{line}}\;$$

##### Cyclooxygenase (COX) Inhibition Activity

Colorimetric assays are frequently used for enzyme analysis because they can be easily adapted to simple microplate formats and need relatively inexpensive and generally available equipment. The use of TMPD could become the method of choice for identifying prospective COX inhibitors [81]. The colorimetric COX (ovine) inhibitor screening assay effectively utilized the peroxidase component of the cyclooxygenase enzyme. Peroxidase activity was assayed colorimetrically by detecting the appearance of TMPD at 590 nm. The activity of COX-1 and COX-2 enzyme inhibitors was determined using a kit provided by Cayman Chemical (Ann Arbor, MI, USA). The kit involved an assay buffer (10X), Heme, COX-1 (Ovine), COX-2 (Ovine), potassium hydroxide, Arachidonic acid, colorimetric substrate and a 96-well plate [82]. The COX inhibition efficacy of the optimized CD-TENs and 6-G was compared with the COX inhibition efficacy of a non-steroidal anti-inflammatory drug (Indomethacin) and that of a natural anti-inflammatory agent (AKBA). It was estimated as the drug concentration that caused 50% enzyme inhibition (IC_50_). The COX-2 selectivity index (SI) was also determined as follows [83]:

(7) $$\left(\mathrm{COX}-2\right)\mathrm{SI}=\frac{\left(\mathrm{COX}-1\right)\mathrm{IC}50\;}{\left(\mathrm{COX}-2\right)\mathrm{IC}50}$$

#### 3.2.11. Statistical Analysis

Statistical analysis of the data was performed by ANOVA and Student’s *t*-test and using SPSS-11 software (SPSS. Inc., Chicago, IL, USA). The results obtained from the 2^3^ factorial design of 6-G-loaded ethoniosomal formulations were analyzed by ANOVA using the Design-Expert software, Version 7.0.0 (Stat-Ease, Inc., Minneapolis, MN, USA) to study the effect of the chosen independent variables on EE% and Q_24h_.

## 4. Conclusions

The present study investigated the development of 6-G-loaded CD-functionalized TENs as a novel ultra-deformable nanocarrier that could enhance the solubility, permeability and stability of 6-G. The 6-G-loaded ENs were formulated according to 2^3^ factorial design, using the ethanol injection technique. The optimized HPβCD-functionalized transethoniosomal formula (F8) was selected on the basis of the highest desirability value. F8 exhibited higher EE%, permeability, deformability and stability than the corresponding TENs, ENs and NVs. Moreover, there was a significant enhancement in both the cytotoxic and anti-inflammatory activity compared to pure 6-G. In brief, these results showed that the synergism between the HPβCD inclusion complex and TENs resulted in development of a promising drug delivery system that can overcome the pitfalls of the poor solubility and limited stability of 6-G.

## Figures and Tables

**Table 1:** Experimental runs, variables and responses of the 2^3^ factorial design for 6-G-loaded ENs.

Formula	Variables				
	Independent			Dependent	
	X1	X2	X3	Y1 *	Y2 *
F1	−1	−1	−1	59.73 ± 1.53	71.20 ± 2.10
F2	−1	1	−1	64.95 ± 2.30	79.29 ± 1.33
F3	−1	−1	1	76.10 ± 1.21	86.64 ± 1.29
F4	−1	1	1	82.43 ± 1.36	92.74 ± 1.54
F5	1	−1	−1	77.63 ± 1.55	64.33 ± 1.29
F6	1	1	−1	82.78 ± 1.03	73.13 ± 1.13
F7	1	−1	1	87.93 ± 2.11	77.55 ± 1.69
F8 #	1	1	1	90.30 ± 1.47	88.05 ± 1.22
Independent variables				Low (−1)	High (+1)
X1: Amount of Span 60 (mg)				350	450
X2: Amount of EA (mg)				0	150
X3: Amount of CD (mM)				0	1

**Notes:** Y1: EE (%), Y2: Q_24h_ (%), * the values are described as mean± SD; *n* = 3, # Optimized ethoniosomal formula. **Abbreviations**: EE, entrapment efficiency; Q24h, % drug released after 24 h; EA, edge activator; CD, cyclodextrin.

**Table 2:** The output results of the 2^3^ factorial design of 6-G-loaded ENs.

Responses	*R* ^2^	Adj. *R*^2^	Pred. *R*^2^	Adequate Precision
EE% (Y1)	0.9545	0.9204	0.8180	14.80
Q_24h_ (Y2)	0.9921	0.9862	0.9685	36.93

**Abbreviations:***R*^2^, the coefficient of determination value; EE%, entrapment efficiency percent of 6-G within ENs; Q_24h_, % 6-G released after 24h; Pred. *R*^2^, predicted *R*^2^; Adj. *R*^2^, Adjusted *R*^2^.

**Table 3:** ANOVA for the 2^3^ factorial design of 6-G-loaded ENs.

Independent Variable	Source	Sum of Squares	df	Mean Square	F-Value	*p*-Value
EE% (Y1)	Model	763.11	3	254.73	27.98	0.0038
	X1	384.06	1	384.06	42.24	0.0029
	X2	45.46	1	45.46	5.00	0.0890
	X3	333.59	1	333.59	36.69	0.0037
Q24h (Y2)	Model	636.6	3	212.20	168.18	0.0001
	X1	89.85	1	89.85	71.21	0.0011
	X2	140.20	1	140.20	111.12	0.0005
	X3	406.55	1	406.55	322.22	<0.0001

**Notes:** Y1: EE (%), Y2: Q_24h_ (%), Amount of Span 60 (X1), Amount of EA (X2), Amount of CD (X3), *p*-value < 0.05 shows that the model terms are significant. **Abbreviation:** SS, the sum of squares; df, the degree of freedom; MS, the mean of squares.

**Table 4:** The kinetic study of the in vitro release of 6-G, 6-G/HPβCD complex and 6-G-loaded ENs.

Formula	Zero Order	First Order	Higuchi Model	Hixson–Crowell	Baker–Lonsdale
F1	0.9548	−0.9844	0.9855	0.9765	0.9900
F2	0.9247	−0.9846	0.9835	0.9691	0.9961
F3	0.8838	−0.9682	0.9591	0.9442	0.9751
F4	0.8606	−0.9808	0.9475	0.9499	0.9810
F5	0.9473	−0.9691	0.9739	0.9627	0.9759
F6	0.9452	−0.9803	0.9830	0.9707	0.9878
F7	0.9513	−0.9891	0.9882	0.9798	0.9931
F8	0.8821	−0.9689	0.9580	0.9441	0.9728
6-G	0.9843	−0.9953	0.9916	0.9962	0.9911
6-G/HPβCD complex	0.9720	−0.9934	0.9912	0.9993	0.9954

**Table 5:** Ex vivo permeation parameters of free 6-G, the optimized 6-G-loaded CD-TENs, TENs, ENs and NVs.

Formula	* J_ss_ (µg cm^−2^ hr ^−1^)	* K_P_ (Cm hr ^−1^)	ER
6-G dispersion	0.54 ± 0.13	0.00054 ± 0.05	-------
CD-TENs	4.65 ± 1.15	0.0046 ± 0.07	8.61
TENs	3.80 ± 1.24	0.0035 ± 0.11	7.20
ENs	3.49 ± 1.13	0.0030 ± 0.14	6.47
NVs	2.77 ± 0.78	0.0021 ± 0.11	5.11

Notes: * Each value denotes the average ± SD (*n* = 3). **Abbreviations:** 6-G, 6-gingerol; CD-TENs, cyclodextrin-functionalized transethoniosomes; TENs, transethoniosomes; EN, ethoniosomes; NVs, niosomes; J_ss_, the steady-state flux; K_P,_ the permeability coefficient; ER, enhancement ratio.

**Table 6:** Effect of storage at 4 °C on the stability of the optimized 6-G-loaded CD-TENs (F8), TENs, ENs and NVs.

Parameter	% Change			
	CD-TENs	TENs	ENs	NVs
Drug content (%)	0.73 ± 0.02	1.25 ± 0.03	1.53 ± 0.02	14.12 ± 0.31
EE (%)	1.21 ± 0.04	2.30 ± 0.08	2.61 ± 0.06	10.83 ± 0.23
Q_24h_ (%)	1.14 ± 0.02	2.83 ± 0.06	2.21 ± 0.04	12.95 ± 0.31

**Notes:** Each value is described as mean ± SD (*n* = 3). **Abbreviations**: 6-G, 6-gingerol; CD-TENs, cyclodextrin-functionalized transethoniosomes; TENs, transethoniosomes; ENs, ethoniosomes; NVs, niosomes; EE, entrapment efficiency; Q24h, % drug released after 24 h.

## Data Availability

The data is contained within the article or Supplementary Materials.

## Supplementary Materials

The following supporting information can be downloaded at: https://www.mdpi.com/article/10.3390/pharmaceutics14061170/s1, Figure S1: The impact of different independent variables; the amount of Span 60, amount of EA and the amount of HPβCD on EE% of 6-G-loaded ENs according to 2^3^ factorial design; Figure S2: The effect of different independent variables; amount of non-ionic surfactant (a), type of non-ionic surfactant (b) and amount of EA (c) on Q_24h_ of 6-G-loaded ENs according to 2^3^ factorial design; Figure S3: Reversed phase C18 silica gel chromatogram of isolated 6-G co-chromatographed with authentic sample (Au 6-G9; S4: ^13^C-NMR (100 MHz, CDCl_3_) of 6-G, carbon multiplicities were determined by APT experiment.; Figure S5: ^1^H-NMR spectrum (400 MHz, CDCl_3_) of 6-G.
