# Supplementary material for: Development of Cyclodextrin-Functionalized Transethoniosomes of 6-Gingerol: Statistical Optimization, In Vitro Characterization and Assessment of Cytotoxic and Anti-Inflammatory Effects
Source: Pharmaceutics. 2022 May 30;14(6):1170. doi: 10.3390/pharmaceutics14061170 (PMC9227240; doi:10.3390/pharmaceutics14061170)
Supplement: Supplementary file 1 [file pharmaceutics-14-01170-s001.zip › pharmaceutics-1705296-supplementary.pdf]

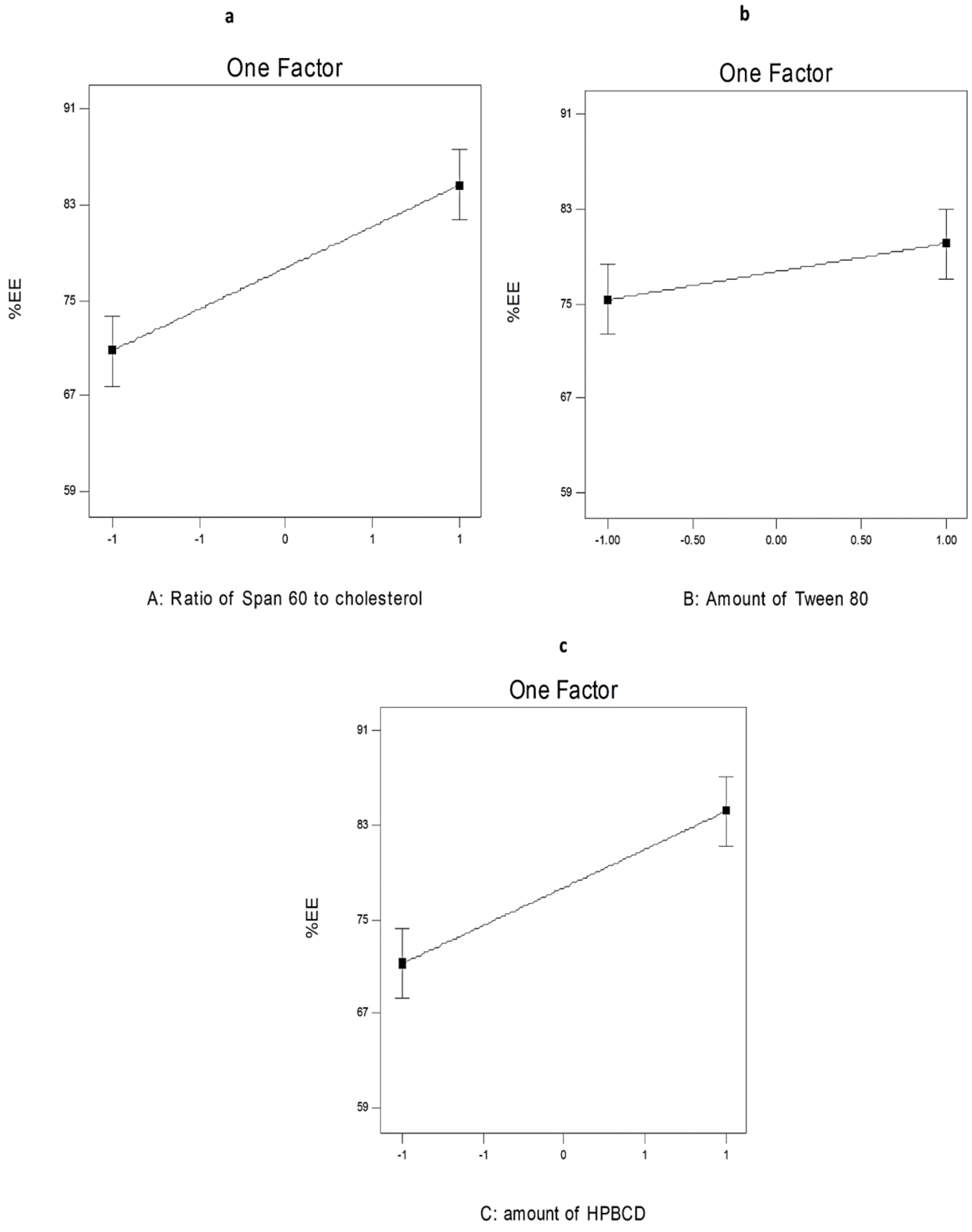

Figure S1. The impact of different independent variables; the amount of Span 60, amount of EA and the amount of HPβCD on EE% of 6-G-loaded ENs according to 2^3 factorial design

Abbreviation: EE, entrapment efficiency of 6-G-loaded ENs; 6-G, 6-gingerol; ENs, ethoniosomes .

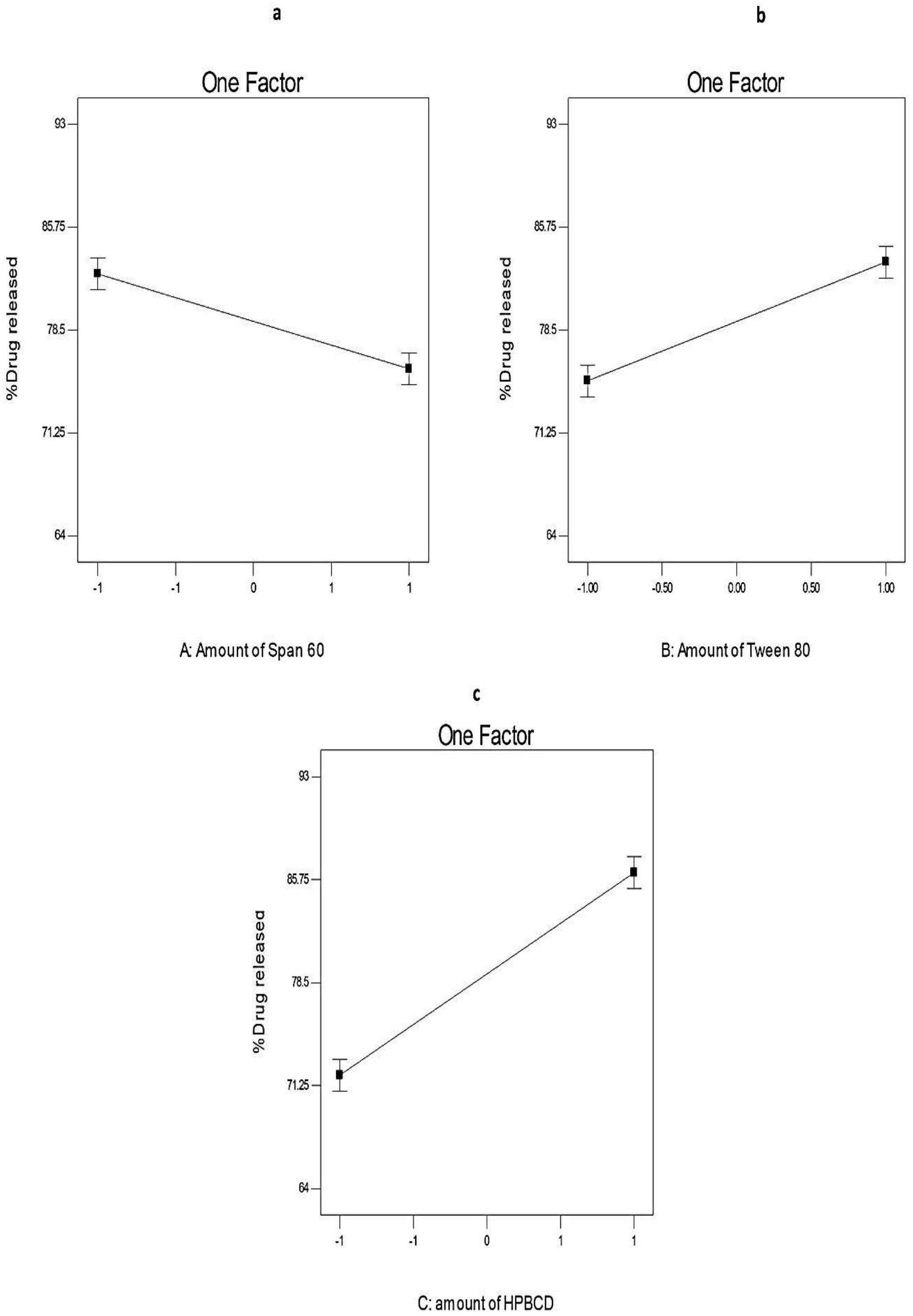

Figure S2. The effect of different independent variables; amount of non-ionic surfactant (a), type of non-ionic surfactant (b) and amount of EA (c) on Q_{24h} of 6-G-loaded ENs according to 2^3 factorial design

Abbreviation: Q_{24h}, % drug released after 24 h; 6-G, 6-gingerol; ENs, ethoniosomes .

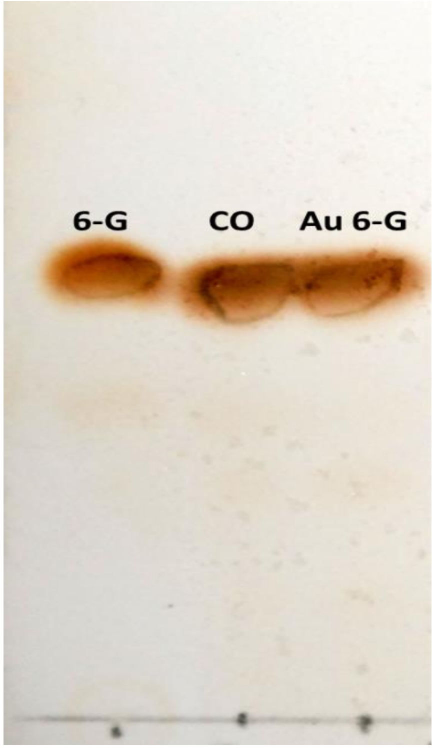

Figure S3. Reversed phase C_{18} silica gel chromatogram of isolated 6-G co-chromatographed with authentic sample (Au 6-G)

Notes: Eluting system was methanol-water (8:2, v/v), visualization using vanillin/sulfuric acid spray reagent, and heating at 110 ºC for 1 min. Abbreviation: 6-G; 6-Gingerol

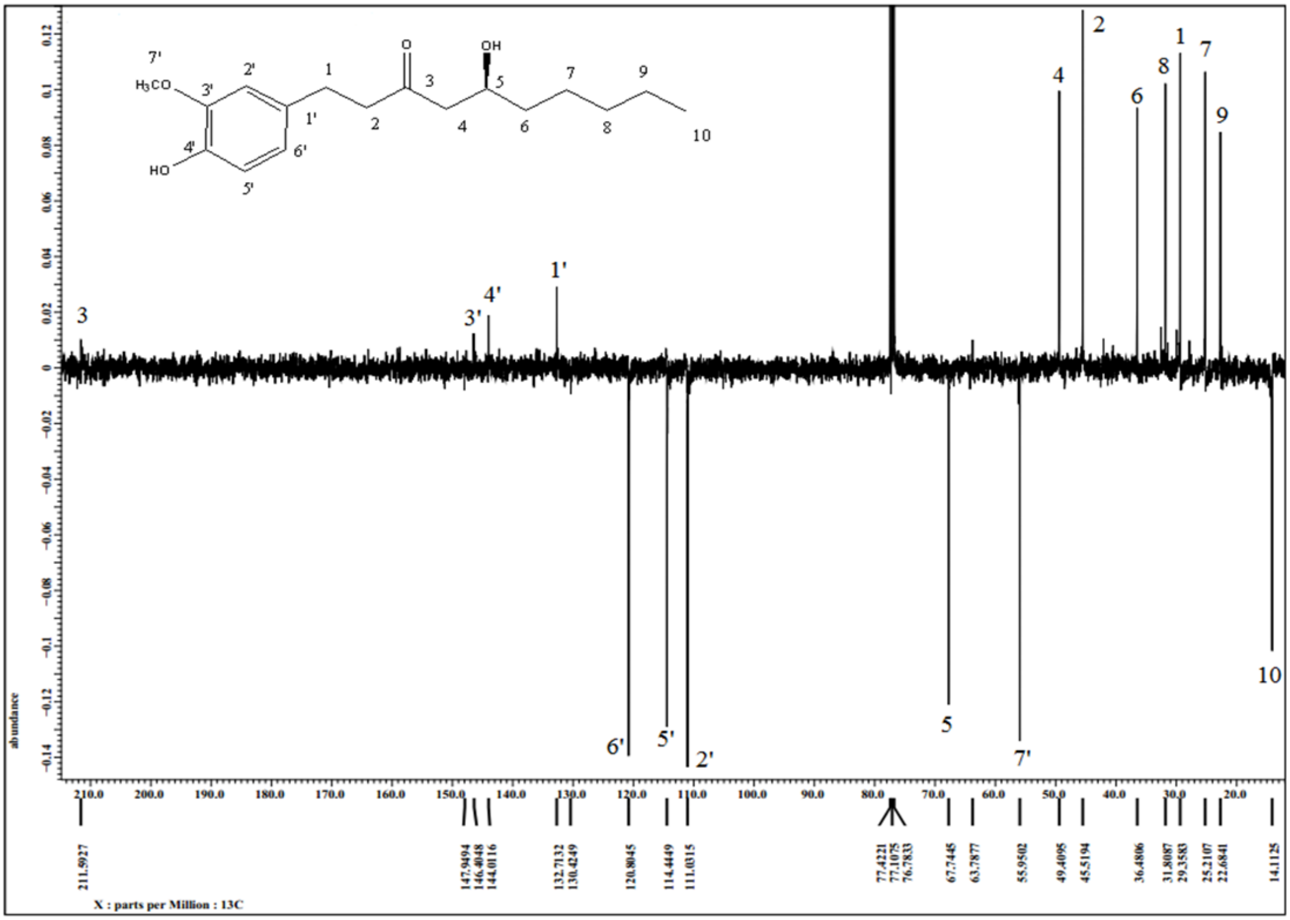

Figure S4. ^{13}C-NMR (100 MHz, $CDCl_3$) of 6-G, carbon multiplicities were determined by APT experiment. Abbreviation: 6-G; 6-Gingerol, ^{13}C-NMR ; Carbon-13 nuclear magnetic resonance $CDCl_3$; Chloroform-d1, APT; Attached proton test

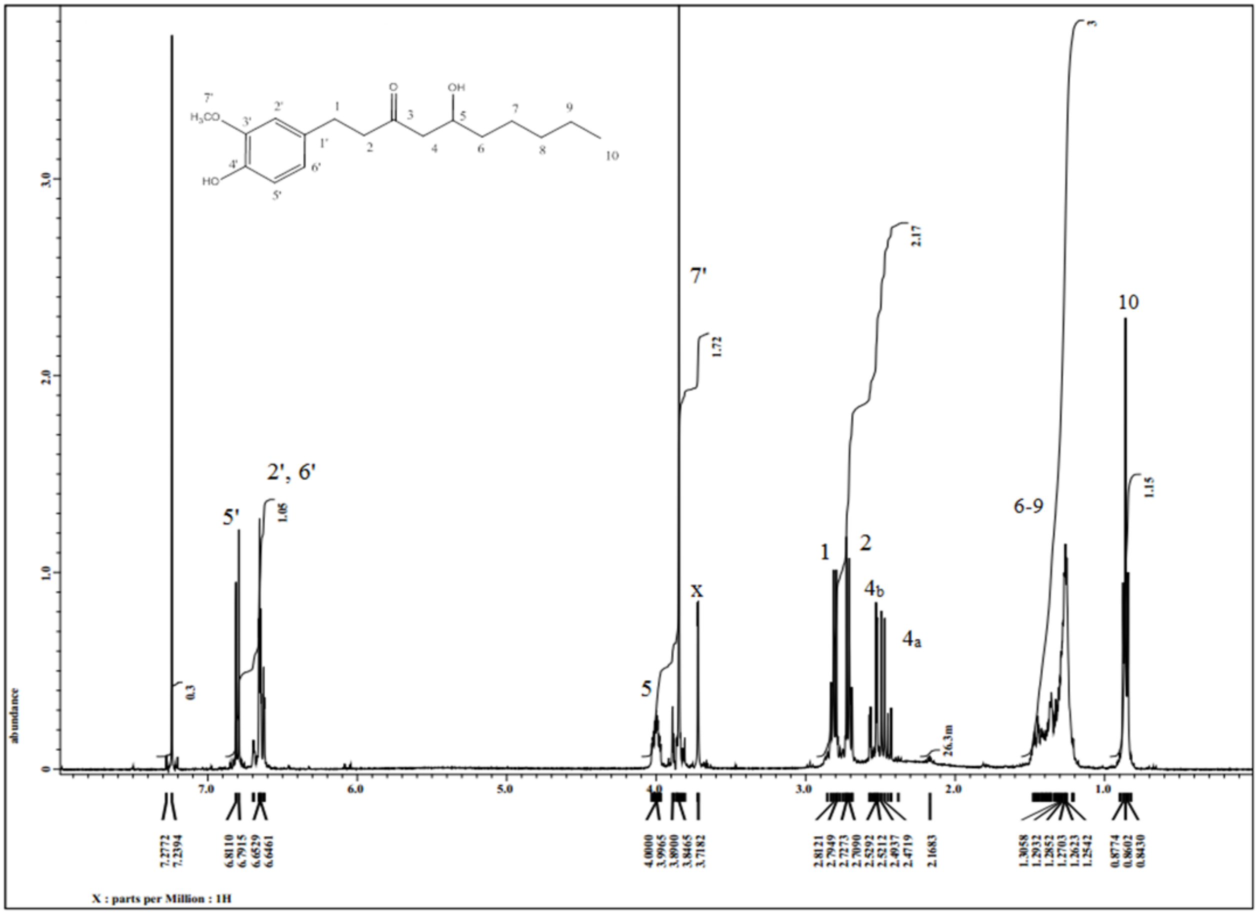

Figure S5. ^{1}H-NMR spectrum (400 MHz, $CDCl_3$) of 6-G.

Abbreviation: 6-G; 6-Gingerol, ^{1}H-NMR spectrum; Proton nuclear magnetic resonance
